# Uncovering mitochondrial dynamics–related genes as potential diagnostic biomarkers for acute myocardial infarction

**DOI:** 10.3389/fcvm.2026.1755024

**Published:** 2026-02-13

**Authors:** Xiaolin Yue, Jinlei Wu, Xueyun Shi, Youshun Xu, Xiaowei Han, Ruijian Li

**Affiliations:** 1 Clinical Research Center for Emergency and Critical Care Medicine of Shandong Province, Qilu Hospital of Shandong University, Jinan, China; 2 Key Laboratory of Emergency and Critical Care Medicine of Shandong Province, Qilu Hospital of Shandong University, Jinan, China; 3 State Key Laboratory for Innovation and Transformation of Luobing Theory, Key Laboratory of Cardiovascular Remodeling and Function Research of MOE, NHC, CAMS and Shandong Province, Department of Cardiology, Qilu Hospital of Shandong University, Jinan, China; 4 Department of Cardiology, Affiliated Hospital of Shandong University of Traditional Chinese Medicine, Jinan, China

**Keywords:** acute myocardial infarction, biomarkers, machine learning, mitochondrial dynamics, regulatory network, single-cell RNA sequencing

## Abstract

**Introduction:**

Mitochondrial dynamics play a vital role in maintaining cardiac energy balance and cellular homeostasis. Increasing evidence suggests that dysregulated mitochondrial dynamics contribute to the development of acute myocardial infarction (AMI). However, the underlying molecular mechanisms and related biomarkers remain largely unclear.

**Methods:**

In this study, transcriptomic profiling of AMI and control samples was used to identify mitochondrial dynamics-associated genes (MD-RGs) linked to AMI progression. Based on the expression of 50 curated MD-RGs, AMI samples were classified into molecular subgroups using single-sample gene set enrichment analysis (ssGSEA). Differentially expressed genes were integrated into multiple machine learning models to identify potential diagnostic biomarkers. Expression validation and receiver operating characteristic (ROC) analyses were performed to assess diagnostic accuracy. Functional enrichment, immune infiltration, and N6-methyladenosine (m6A) regulator correlation analyses were conducted to explore biological mechanisms. Key cell types were identified through single-cell RNA sequencing (scRNA-seq) analysis, and biomarker expression was validated by reverse transcription quantitative PCR (RT-qPCR) in patient-derived samples.

**Results:**

Two genes, COX7B and SNORD54, were identified as novel biomarkers associated with mitochondrial dynamics in AMI. ROC and nomogram analyses confirmed their strong diagnostic performance. Enrichment analysis revealed shared pathways including oxidative phosphorylation and Notch signaling, while six m6A regulators (HNRNPC, KIAA1429, METTL3, WTAP, YTHDC1, and YTHDC2) were markedly downregulated, suggesting possible epigenetic involvement. RT-qPCR confirmed reduced expression of COX7B and SNORD54 in AMI tissues. Single-cell analysis further identified monocytes and natural killer (NK) cells as key cell types linked to these biomarkers.

**Discussion:**

Collectively, this study identifies COX7B and SNORD54 as mitochondrial dynamics-related biomarkers and highlights the role of monocytes and NK cells in AMI, offering new insight into mitochondrial dysfunction-driven cardiac injury and potential targets for precision diagnosis and therapy.

## Introduction

Acute myocardial infarction (AMI), a leading cause of death and chronic heart failure (HF) worldwide, occurs when prolonged ischemia causes cardiomyocytes death (1). These results in progressive decline in heart function, potentially leading to HF and life-threatening arrhythmias. The most common cause of AMI is atherothrombotic coronary artery disease, where unstable atherosclerotic plaques rupture or erode, triggering thrombus formation that blocks the coronary artery or causes distal embolization (2). In cases of AMI, early and successful revascularization can prevent the loss of contractile heart muscle, reduce infarct size, and improve clinical outcomes (3). However, reperfusion can paradoxically cause additional damage to the heart, known as myocardial ischemia-reperfusion (I/R) injury (4). Studies have shown that I/R injury involves various forms of cell death, including apoptosis, necrosis, pyroptosis, and autophagy-associated cell death (5). To address this, new therapies are needed to protect the heart from the harmful effects of I/R injury, minimize infarct size, preserve left ventricular (LV) function, and prevent the development of heart failure (HF) (6).

Mitochondrial dynamics is a biological process in order to maintain normal physiological functions, mainly including mitochondrial fusion, fission and mitochondrial autophagy (7). As one of the most abundant organelles in cardiomyocytes, mitochondria play an important role in various physiological processes, including cardiomyocytes proliferation, apoptosis and signal transduction (8). Mitochondrial dysfunction is a pivotal pathological basis for myocardial I/R injury and it represents a critical therapeutic target for the mitigation of myocardial damage (9, 10). However, the exact pathogenesis and molecular mechanism of mitochondrial dynamics in AMI have not been fully elucidated.

In AMI, mitochondrial dysfunction occurs due to hypoxia and oxidative stress, leading to energy metabolism disturbances and cell death. Genes such as peroxisome proliferator-activated receptor gamma co-activator 1 alpha (PGC-1*α*), optic atrophy 1 (Opa1), mitofusin-2 (MFN2) and dynamin-related protein 1 (Drp1) regulate mitochondrial biogenesis, morphological changes, and autophagy in the heart, thereby affecting cardiac function (11). The increase in mitochondrial fission is accompanied by a decrease in mitochondrial fusion, as evidenced by the upregulation of Drp1 and downregulation of Opa1, both of which lead to cardiac damage (12). Although existing research has highlighted the importance of mitochondria in AMI, certain aspects remain insufficiently addressed, including the specific gene regulatory mechanisms, the relationship between changes in mitochondrial function and myocardial injury. Moreover, the insufficient sensitivity of existing markers leads to delayed diagnosis or poor therapeutic effect. We will target specific genes for in-depth study of mitochondrial dynamics to provide new insights into the pathophysiology of AMI, which will help develop new therapeutic targets for AMI.

To explore the potential involvement of mitochondrial dynamics in AMI, this study procured AMI-associated datasets and identified 50 mitochondrial dynamics-related genes (MD-RGs) from publicly available databases. Utilizing bioinformatics approaches, we conducted a screening for candidate biomarkers linked to AMI. Additionally, we performed Gene Set Enrichment Analysis (GSEA) for functional enrichment, alongside assessments of immune cell infiltration and single-sample Gene Set Enrichment Analysis (ssGSEA) of immune functions. To identify the key cell populations driving the biological processes, we performed single-cell RNA sequencing (scRNA-seq). This high-resolution technique allowed us to analyze the transcriptomes of individual cells, revealing distinct cell types and states within the tissue. Using computational tools such as clustering and differential gene expression analysis, we identified specific cell subsets with unique functional roles that were central to the mechanisms in the context of AMI.

## Material and methods

### Data collection

Data on AMI-related gene expression matrices were searched by gene expression omnibus (GEO). We selected patients with MI for 6 months. Specifically, GSE62646 dataset was based on GPL6244 platform, containing blood samples from 28 AMI patients (6 months) and 14 patients with stable coronary artery disease without a history of myocardial infarction (MI). On the other hand, GSE59867 dataset contained blood samples from 83 AMI patients (6 months) and 46 patients with stable coronary artery disease without a history of MI, and the sequencing platform was GPL6244. In the GSE62646 and GSE59867 datasets, blood samples collected on non-first-day-of-admission were excluded to ensure that the selected disease samples were not influenced by treatment. We considered patients with stable coronary artery disease without a history of MI as the control group for this study. Meanwhile, the single cell dataset GSE269269 included peripheral blood mononuclear cells (PBMC) of 5 AMI patients without plaque rupture. Mitochondrial dynamics, which encompasses processes like mitochondrial fission, fusion, and autophagy, is essential for crucial role in cellular energy metabolism and maintenance of homeostasis. To comprehensively analyze these processes, 15 genes closely related to mitochondrial fission and 9 genes associated with mitochondrial fusion were screened using the MitoCarta 3.0 database. Additionally, 28 genes related to mitophagy were retrieved from the Reactome database. After merging and eliminating duplicates, a final set of 50 MD-RGs was identified (Supplementary Table S1). These genes constitute a valuable resource for in-depth investigations into the roles of mitochondrial dynamics in cellular physiology and pathology.

### Differential expression analysis

Based on a pre-screened set of (MD-RGs), we employed the single-sample gene set enrichment analysis (ssGSEA) method from the GSVA package (v 1.42.0) (13) to calculate the enrichment scores of MD-RGs for each AMI sample and control sample in the GSE62646 dataset, aiming to reflect the overall activity level of this gene set in individual samples and to compare the differences in scores between the two groups. To further explore the heterogeneity of mitochondrial dynamics states among AMI samples, we stratified all AMI samples into a high-score group and a low-score group based on the median value of their MD-RGs scores (14). Subsequent differential expression analysis was undertaken via limma package (v 3.54.1) with the thresholds of *P* < 0.05 and |log2 Fold Change (FC)| > 0.5, aiming to identify differentially expressed genes (DEGs) between AMI and control samples as well as between high- and low-scoring groups (15). Volcano maps and heat maps were created with the ggplot2-package (v 3.3.6) (16) and heatmap-package (v 1.0.12) (17), respectively, to demonstrate DEGs expression patterns. Next, overlapping analysis of the two sets of DEGs was completed to determine the common DEGs.

### Functional annotation and protein-protein interactions (PPI) analyses

To reveal the biological functions that common DEGs were involved in the development of AMI, Gene Ontology (GO) annotation and Kyoto Encyclopedia of Genes and Genomes (KEGG) pathway enrichment analyses were undertaken via cluster Profiler-package (v 4.6.2) (18) and the org.Hs.eg.db-package (v 3.16.0) (19) (*P* < 0.05). In addition, a PPI network between common DEGs was created through STRING (confidence level >0.15) to elucidate the association between them at the protein level.

### Machine learning algorithms

Machine learning, a branch of artificial intelligence, focuses on empowering computer systems to learn from data and improve performance through the use of statistical techniques, with a wide and diverse range of applications in the biomedical field (20). In this study, we utilized two machine learning algorithms to identify feature genes closely associated with AMI from common DEGs in GSE62646 dataset. At first, the glmnet-package (v 4.1-4) (21) was utilized to perform least absolute shrinkage and selection operator (LASSO) analysis, with the parameter “family” set to binomial and “lambda” set to 0. This approach was considered as the optimal method for selecting feature genes. Secondly, Boruta algorithm, implemented through the Boruta-package (v 8.0.0), was based on the idea of random forests, where the importance of each feature was evaluated by comparing it with randomly generated “shadow” features (22). During the screening process using the Boruta algorithm, the program defined DEGs, categorizing them into accepted and rejected groups. The accepted genes represented the feature genes selected by the Boruta model. After that, the intersection of two sets of feature genes was extracted with the help of Venndiagram-package (v 1.7.3), which were recorded as candidate genes (23).

### Expression analysis and receiver operating characteristic (ROC) analysis

In GSE62646 and GSE59867 datasets, the expression trends of candidate genes were further compared between AMI and control samples utilizing the Wilcoxon test. Our focus was on genes exhibiting stable expression, characterized by consistent trends across both datasets and significant group differences (*P* < 0.05). Further, to assess the diagnostic accuracy of the model, ROC curves were generated based on gene expression in both datasets with the use of the ROC-package (v 1.18.0), and genes with the area under curve (AUC) greater than 0.7 were defined as biomarkers associated with mitochondrial dynamics in AMI (24). In general, the AUC greater than 0.7 indicated that the gene was more effective in diagnosing the disease. Subsequently, the biomarkers were input into Gene MANIA online website to generate a network, aiming to explore potential mechanisms of biomarkers in AMI.

### Creation and assessment of nomogram

To predict the occurrence of AMI from perspective of the biomarkers as a whole, we integrated the expression of the biomarkers and subsequently constructed a nomogram in GSE62646 dataset applying the rms-package (v 6.5-0) (25). Furthermore, calibration curve and ROC curve were created to estimate the accuracy of nomogram predictions, as well as decision curve analysis (DCA) was completed to determine the likelihood of clinical benefit from the nomogram.

### Enrichment analysis

GSEA was conducted to identify gene sets associated with specific biological processes, pathways, or functions related to biomarkers. The KEGG gene set (c2.cp.kegg.v7.4.symbols.gmt) retrieved from MsigDB database served as the background gene set, and gene set enrichment analysis (GSEA) was implemented with cluster Profiler-package. Briefly, based on all samples from GSE62646, Spearman correlation coefficients between each biomarker and the remaining genes were computed by the psych-package (v 2.1.6), followed by ranking the genes according to the coefficients (from highest to lowest). Then, the ranked genes were treated as the gene set to be detected, and the GSEA was then completed. The gene set with *P* < 0.05 and q < 0.25 was considered significant.

### Immunological characterization

To explore the immune profile in AMI, CIBERSORT method was employed to infer the relative proportions of 22 immune-infiltrating cells in each sample for GSE62646 dataset (26, 27). Next, differences in proportions of immune-infiltrating cells between AMI and control groups were compared by Wilcoxon test (*P* < 0.05), followed by drawing box plot using ggplot2-package to visualize the results. Following this, Spearman correlation analysis was completed between the differential immune cells as well as between the differential immune cells and biomarkers to explore their associations, with thresholds set at |cor| > 0.3 and *P* < 0.05.

### Construction of molecular regulatory network

To elucidate the molecular regulatory mechanisms of the biomarkers, microRNAs (miRNAs) regulating the biomarkers were predicted by applying the miRDB and TargetScan databases. Next, starBase and miRNet databases were utilized to retrieve lncRNAs that bound to the miRNAs obtained as described above. In addition, transcription factors (TFs) targeting biomarkers were collected through accessing University of California, Santa Cruz Genome Browser (UCSC). By integrating the relationship pairs obtained above, the regulatory networks of lncRNA-miRNA-mRNA and TF-biomarker were created via Cytoscape software (v 3.9.0) (28).

### Correlation analysis of m6A regulators with biomarkers

The m6A regulators are crucial for post-transcriptional gene expression regulation, which in turn affects a series of physiological and pathological functions. Related research has emphasized that m6A can participate in regulating the development of AMI (29). Therefore, this study used the Wilcoxon test to compare expression differences of 13 widely reported m6A regulators between AMI and control groups in GSE62646 dataset (*P* < 0.05). The 13 m6A regulators were METTL3, METTL14, WTAP, KIAA1429, RAMI15, ZC3H13, YTHDC1, YTHDC2, YTHDF1, YTHDF2, HNRNPC, FTO, and ALKBH5. Thereafter, Spearman correlation analysis was carried out for biomarkers and m6A regulators in AMI samples using the psych-package (*P* < 0.05).

### Disease and drug prediction and molecular docking

AMI is the most common public health cardiovascular disease with a high number of complications. Therefore, diseases significantly associated with biomarkers (top 20) were analyzed using comparative toxicogenomics database (CTD), and the results were visualized by Cytoscape. In addition, small molecule drugs targeting biomarkers were predicted by thorough analysis of the DGIdb database. Subsequent molecular docking was accomplished to investigate the feasibility of treating AMI. Specifically, it was possible to obtain the 3D structures of small-molecule medications applying PubChem database. Next, Protein Data Bank (PDB) database was applied to acquire the protein crystal structures that corresponded to the biomarkers. Ultimately, molecular docking procedure was executed by applying CB-Dock2, and docking binding energy was calculated. Docking results were generally considered feasible for docking binding energies less than −5 kcal/mol.

### Identification of the key cells by scRNA-Seq analysis

The data of AMI samples from the GSE269269 dataset were filtered for scRNA-seq analysis by the “Seurat” package (v 5.1.0) (30). Specifically, the criteria for filtered were that (1) low quality cells with genes less than 200 and abnormally highly expressed cells with genes greater than 5,000; (2) the sum of the expression of all genes gauged for per cell was ≥25,000 or <500; (3) cells with percentage of mitochondria greater than 25%. In order to obtain highly variable genes (HVGs), the above filtered data were normalized by the NormalizeData function in “Seurat” package (v 5.1.0) to obtain the standardized data. Then, the FindVariableFeatures function was carried out to select the top 2,000 HVGs. The result was visualized and the top 10 genes with highest intercellular expression variation were labeled by the LablePoints function. To identify the statistically significant principal components, data scaling was carried out with ScaleData function in “Seurat” package (v 5.1.0). After that, a principal component analysis (PCA) plot was drawn between all the samples to confirm whether the overall distribution of cells was consistent per sample and whether significant outlier samples existed. At the same time, PCA was also conducted using these 2,000 HVGs. Then, JackStrawPlot function served to contrast the *p*-values distribution of each principal components (PCs) (*p* < 0.05). The scree plot was visualized by applying the ElbowPlot function, and the number of PCs corresponding to where the curve began to level off were selected for uniform manifold approximation and projection (UMAP) cell cluster analysis (resolution = 0.8). Furthermore, use the FindAllMarkers function in “Seurat” package (v 5.1.0) to identify the marker genes in each cell cluster of the single—cell dataset (logfc.threshold = 0.5, min.pct = 0.25, return.thresh = 0.01). Then, annotating different cell clusters to different cell types based on the marker genes using the “singleR” package (v 2.4.0) (31) and the CellMarker database. The bubble plots depicting different marker gene expression in different cell types were drawn. Finally, to identify key cells associated with key genes, UMAP plots, bubble plots and scatter plots were created to show biomarkers expression on all cell types in AMI samples. The cells in which the expression levels of biomarkers were relatively high and had large cell number of cells were defined as key cells.

### Cell communication and pseudo-time analysis

Cell communication analysis was conducted to examine the expression and pairing of receptors and ligands in different cells, aiming to infer interactions between different cells. The communication network between cells were analyzed by the “CellChat” package (v 1.6.1) (32). To gain deeper insights into heterogeneity of the key cells, based on key cells, in GSE269269, the key cells were regrouped, the cells were annotated into different cell subsets, and the clustering results were visualized using UMAP. In order to investigate the differentiation trajectory and evolution of key cells during development, the “monocle” package (v 2.26.0) (33) was utilized for pseudo-time analysis to investigate alterations in the developmental trajectory of key cells and variations in the expression of biomarkers.

### Reverse transcription quantitative PCR (RT-qPCR)

RT-qPCR was performed to detect and analyze the expression levels of specific mRNAs in blood samples.The study was conducted in accordance with the Declaration of Helsinki, and RNA was collected from 5 pairs of blood samples, including 5 acute myocardial infarction samples and 5 control samples. Consistent with the previous data, we selected patients with myocardial infarction for 6 months. According to the corresponding age and gender, healthy people were selected as controls. The agency responsible for ethical approval was designated as Qilu hospital. The participants all completed and signed an informed consent form, and the ethical approval agency was Ethics Committee of Qilu Hospital of Shandong University [approval no. KYLL-2022(ZM)-082]. Since patient genetic data are involved, we kept patient-specific information confidential. Whole blood samples were collected from patients using EDTA-anticoagulant tubes. Total RNA was extracted from whole blood samples using the RNAprep Pure Hi-Blood Kit (DP443, Tiangen Biotech, Beijing, China). Briefly, erythrocytes were lysed, and leukocytes were homogenized in a proprietary lysis buffer. After ethanol addition, the lysate was applied to a silica-membrane column for RNA binding. The column was washed, treated with DNase I to remove genomic DNA contamination, washed again, and total RNA was finally eluted in RNase-free water. RNA concentrations were measured by NanoPhotometer N50. Secondly, mRNA was transcribed to synthesize cDNA using SweScript First Strand cDNA synthesis kit (Servicebio, Wuhan, China). For Quantitative RT-PCR analysis, each reaction mixture consisted of 3 µL cDNA, 5 µL 2x Universal Blue SYBR Green qPCR Master Mix (Servicebio), 1 µL forward primer (10 µM), and 1 µL reverse primer (10 µM) (Supplementary Table S2). The mRNA levels were normalized by GAPDH which was selected as an endogenous reference gene. The relative gene expression was calculated by 2-^△△^Ct, and *p*-value was calculated by Graphpad Prism 5.

### Statistical analysis

Group comparisons were assessed using the Wilcoxon test. Statistical discrepancies were recognized as *P*-value below 0.05. In this work, bioinformatic statistical analyses were implemented employing R program (version 4.2.3).

## Results

### Identificationg of 29 common DEGs associated with mitochondrial dynamics in AMI

**Figure 1:**
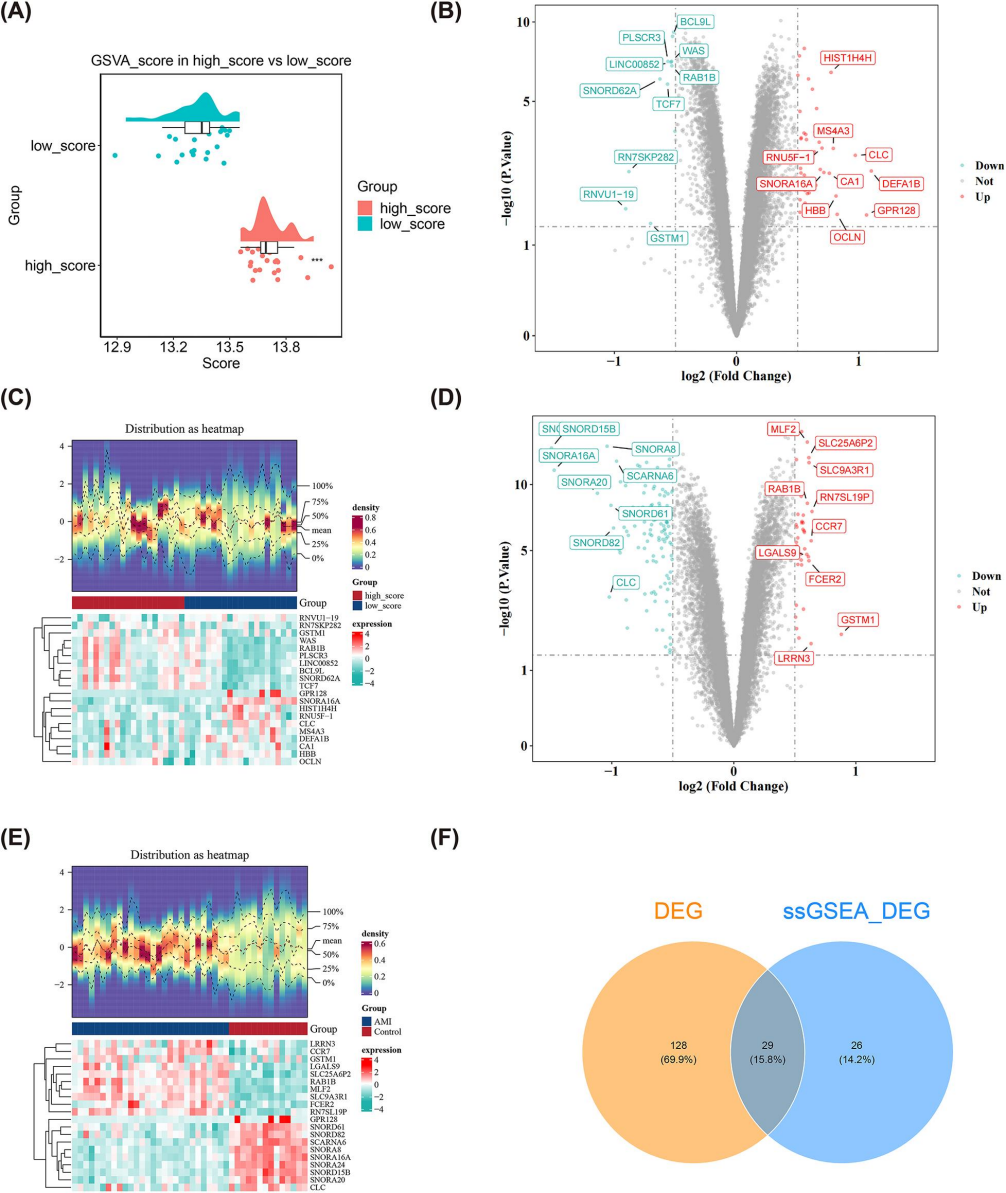
Screening for AMI mitochondrial dynamics-related differential genes. **(A,B)** GSE62646 dataset analysed with ssGSEAs. **(C,D)** Volcano and heat maps of differential genes in the high scoring group vs. the scoring group. **(E,F)** Volcano and heat maps of differential genes in Volcano and heat maps of differential genes in AMI and control samples in the GSE62646 dataset. **(G)** Venn diagram of DEGs in high and low scoring groups and GSE62646 dataset.

Within the GSE62646 dataset, differential analysis revealed a significant decrease in MD-RGs scores in AMI samples compared to control samples (*p* < 0.001) (Figure 1A). This might suggest that the expression of MD-RGs may influence the progression of disease in patients with AMI. In the AMI sample for the GSE62646 dataset, there was a marked discrepancy in MD-RGs scores between high- and low-scoring groups divided based on the ssGSEAs algorithm (*P* < 0.05) (Figure 1B). By setting thresholds of *P* < 0.05 and |log2FC| > 0.5, 55 DEGs (43 up-regulated and 12 down-regulated in high-scoring groups) between high- and low-scoring groups (Figures 1C,D), as well as 157 DEGs (118 up-regulated and 39 down-regulated in AMI samples) were mined between AMI and control samples in GSE62646 dataset (Figures 1E). Following this, the Venn diagram displayed 29 common DEGs by overlapping the two sets of DEGs (Figure 1F). These 29 genes likely represented a gene set that played roles in both the occurrence of AMI and different mitochondrial functional states.

### Revealing the biological functions and PPI of 29 common DEGs

**Figure 2:**
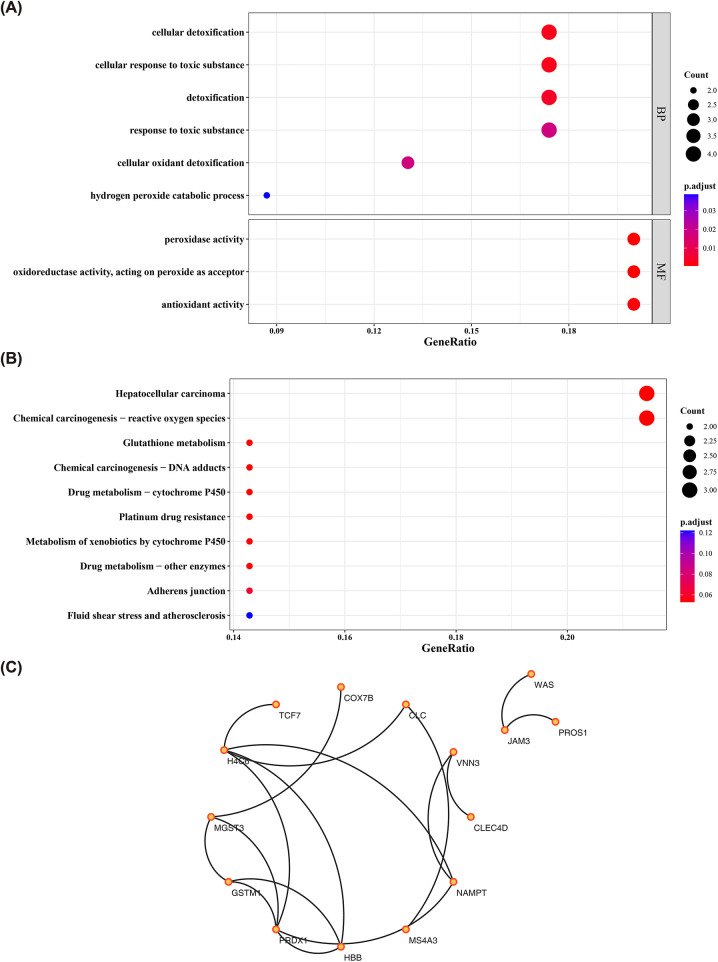
The biological functions analysis DEGs. **(A)** Bubble plot of GO functional enrichment analysis for DEGs. The *x*-axis “GeneRatio” represents the proportion of differentially expressed genes in the corresponding GO term relative to the total number of genes in that term. The *y*-axis shows the names of the enriched GO terms. The bubble color corresponds to the adjusted *P*-value (p.adjust) of the term; a redder color indicates a higher enrichment significance. The bubble size represents the number of differentially expressed genes (Count) under that term; a larger bubble indicates a greater number of differentially expressed genes; **(B)** Bubble plot of KEGG pathway enrichment analysis for DEGs. The *x*-axis “GeneRatio” represents the proportion of differentially expressed genes mapped to a given pathway relative to the total number of genes annotated to that pathway. The *y*-axis lists the names of the significantly enriched KEGG pathways; **(C)** The PPI network of the DEGs.

These 29 common DEGs were enriched and analyzed, yielding 9 GO entries and 13 KEGG signaling pathways (*P* < 0.05). With respect to GO, the entries were mainly related to “cellular detoxification”, “detoxification”, “response to toxic substance”, “cellular oxidant detoxification”, “hydrogen peroxide catabolic process”, “peroxidase activity”, and so on (Figure 2A, Supplementary Table S3). KEGG analysis elucidated that candidate genes were engaged in “Chemical carcinogenesis—reactive oxygen species”, “Glutathione metabolism”, “Drug metabolism—cytochrome P450”, etc. (Figure 2B, Supplementary Table S4). In addition, the PPI network of the 29 common DEGs, containing 15 points and 17 edges, was illustrated in Figure 2C, and it could be noted that H4C6, PRDX1, and NAMPT seemed to have stronger interactions with the remaining genes. This suggested that these genes might occupy pivotal positions in co-regulated biological processes and merited further investigation.

### Identification of candidate genes associated with AMI

**Figure 3:**
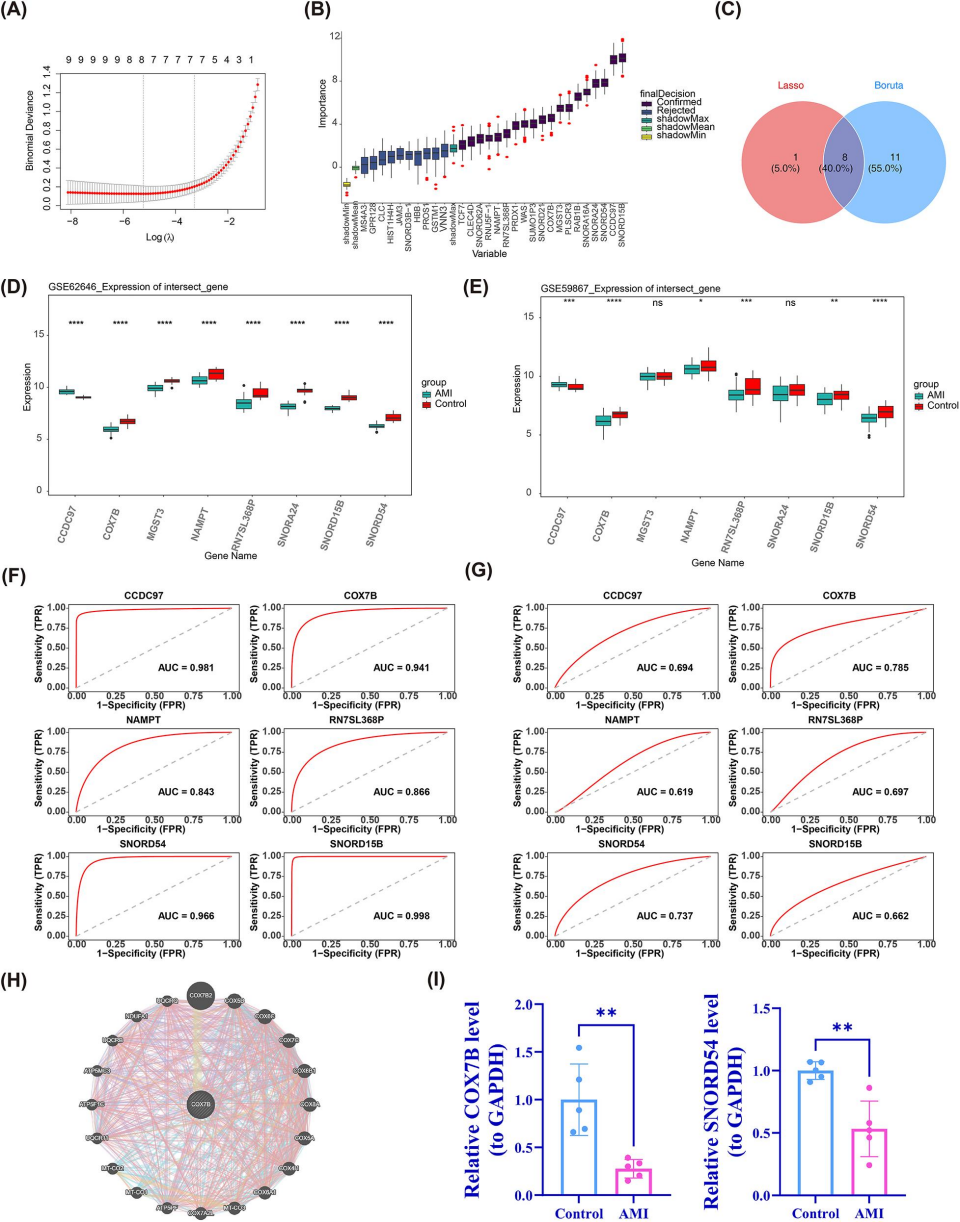
Identification of candidate genes associated with AMI. **(A)** Genes associated with AMI analyzed with LASSO algorithm; **(B)** Genes associated with AMI analyzed with Boruta algorithm; **(C)** Venn diagram of common genes in the LASSO algorithm and Boruta algorithms; **(D,E)** The markedly distinct genes between AMI and control groups in GSE62646 and GSE59867 datasets; **(F,G)** The ROC curve of candidate genes associated with AMI; **(H)** The interaction network between COX7B and highly related genes; **(I)** Quantitative RT-PCR analysis of COX7B and SNORD54 RNA levels (*n* = 5). **P < 0.01 vs. Control.

These 29 common DEGs were incorporated into two machine learning algorithms to identify feature genes. The LASSO algorithm identified nine feature genes associated with AMI at lambda. min = 0.000299114, namely SNORA24, SNORD15B, CLC, RN7SL368P, SNORD54, COX7B, NAMPT, MGST3, and CCDC97 (Figure 3A). Furthermore, Boruta algorithm screened out 19 feature genes based on each feature's importance, as shown in Figure 3B. We utilized a Venn diagram to identify common genes in the two algorithms, yielding a total of eight genes (SNORA24, SNORD15B, RN7SL368P, COX7B, NAMPT, MGST3, CCDC97, and SNORD54), which were recorded as candidate genes for further exploration (Figure 3C). Given this, the integration of two machine learning algorithms effectively narrowed down the range of key genes, thereby enhancing the focus of subsequent analyses.

### Screening and diagnostic value of COX7B and SNORD54 in AMI

The expression analyses of candidate genes were conducted in GSE62646 and GSE59867 datasets, which showed that six genes were markedly distinct between AMI and control groups (*P* < 0.05) (Figures 3D,E). Among them, CCDC97 was up-regulated in AMI samples, while the remaining five genes (COX7B, NAMPT, RN7SL368P, SNORD54, and SNORD15B) were down-regulated in AMI samples. What's more, the AUC values of two (COX7B and SNORD54) of these six genes in both datasets were greater than 0.7 through the ROC curves (Figures 3F,G), implying that, they could better differentiate between AMI and control samples. Therefore, COX7B and SNORD54 were considered as biomarkers associated with mitochondrial dynamics in AMI. In addition, an interaction network between COX7B and 20 highly related genes was generated on the Gene MANIA platform, with 8.01% co-expression, 77.64% physical interactions, 5.37% prediction, 3.63% co-localization, 2.87% genetic interactions, 1.88% pathways, and 0.60% shared protein structural (Figure 3H). Unfortunately, the interaction network of SNORD54 was not predicted. Finally, we estimated the expression of 2 biomarkers by RT-qPCR. The expression levels of COX7B and SNORD54 were significantly downregulated in peripheral blood mononuclear cells (PBMCs) of AMI samples compared to control (*P* < 0.05), consistent with its expression in GSE62646 and GSE59867 (Figure 3I). In summary, both COX7B and SNORD54 demonstrated stable differential expression at the transcriptional level and in clinical samples, along with good diagnostic differentiation capability, establishing a foundation for their potential as biomarkers for AMI.

### Building an effective nomogram for diagnosing AMI

**Figure 4:**
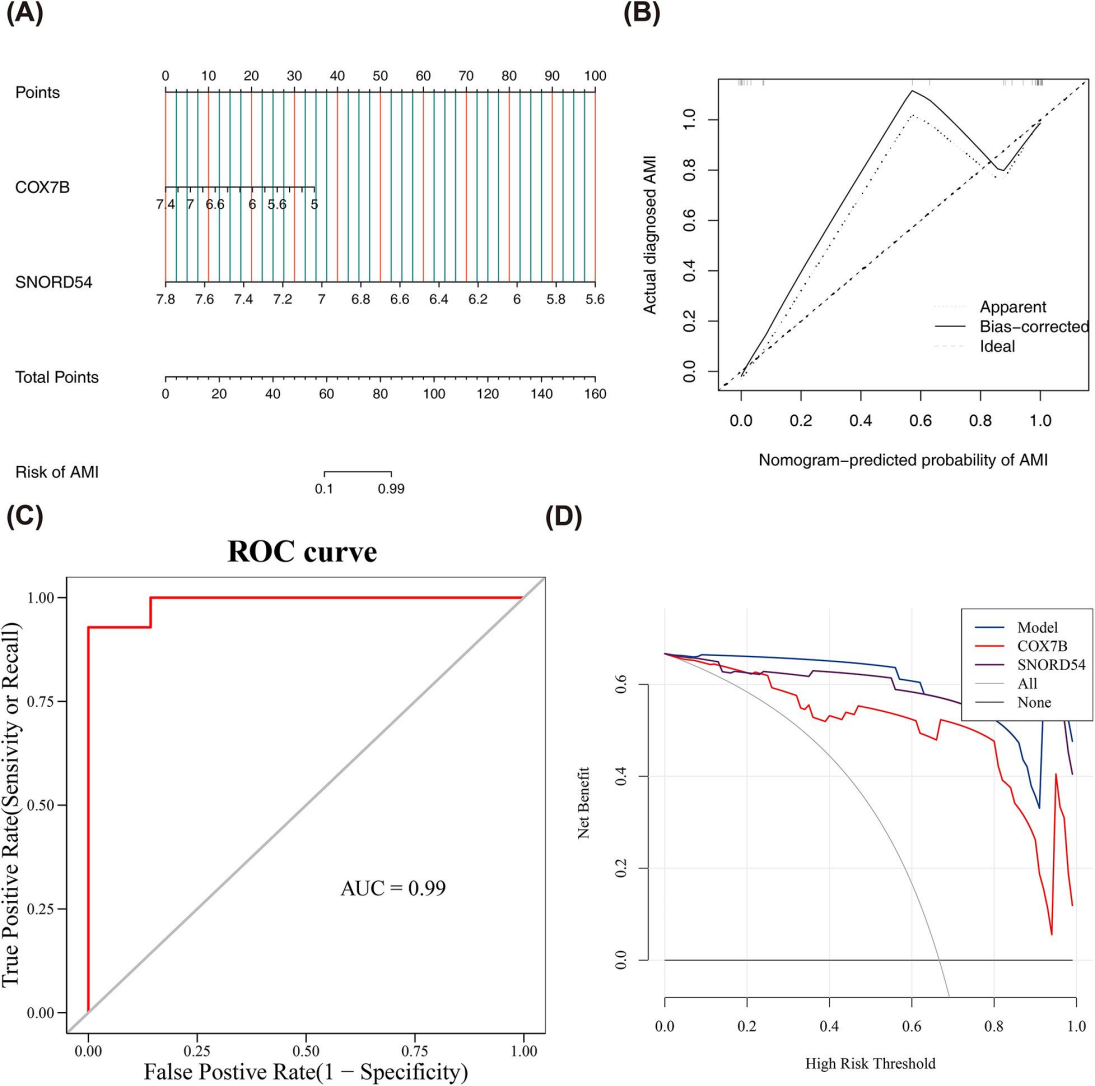
Effective nomogram for diagnosing AMI. **(A)** Nomogram model in GSE62646 dataset; **(B,C)** The calibration curve and ROC curve shows that the accuracy of the nomogram; **(D)** The Decision Curve Analysis of nomogram.

We created a nomogram model in GSE62646 dataset so as to facilitate the clinical prediction of AMI using the selected two biomarkers (Figure 4A). In the nomogram, the lower the expression of the two biomarkers and the higher the total score, the higher the risk of developing AMI. The slope of the calibration curve was close to 1 and AUC value of ROC curve was 0.99, both of which indicated that the accuracy of the nomogram in predicting AMI was superior (Figures 4B,C). The DCA results revealed that the nomogram yielded a net benefit superior to that of the individual biomarker (Figure 4D). In conclusion, nomogram integrated by two biomarkers exhibited high potential clinical value.

### Elucidating the biological mechanisms of biomarkers

**Figure 5:**
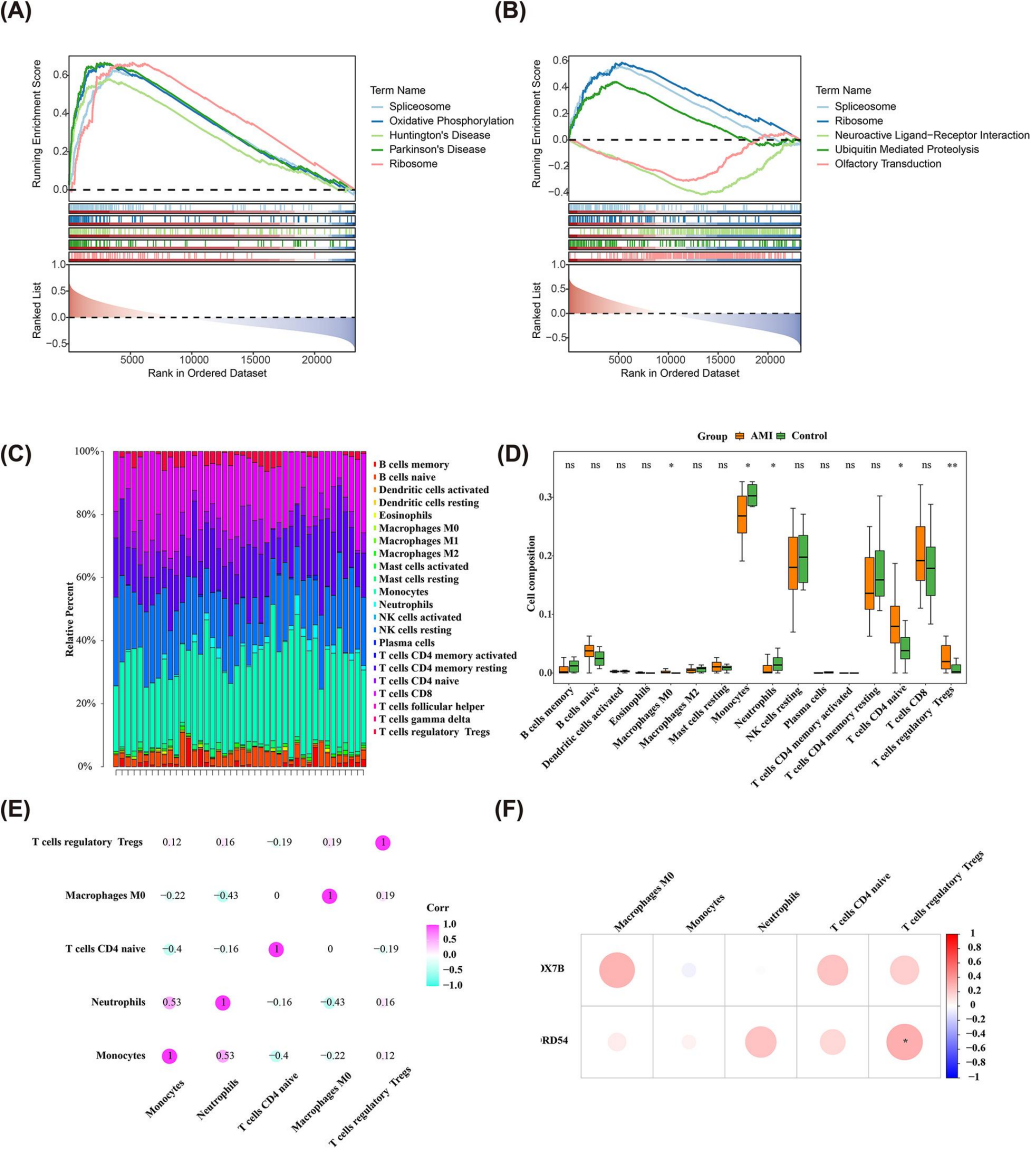
Biological mechanisms of AMI biomarkers. **(A,B)** KEGG enrichment analyses on the two biomarkers in AMI and the top 5 pathways; **(C)** CIBERSORT analysis of immune cells in GSE62646 dataset; **(D)** Box plots of differential expression of immune cells in AD and controls in the training set; **(E)** Heatmap of the correlation between the 5 differential immune cells in the disease samples of the training set; **(F)** Correlation between biomarkers and differential immune cells.

In order to elucidate the biological mechanisms of two biomarkers in AMI, we completed GSEA in the GSE62646 dataset. Results indicated that the expression of two biomarkers was linked to “calcium signaling pathway”, “oxidative phosphorylation”, “MAPK signaling pathway”, “Wnt signaling pathway”, “cell cycle”, “Notch signaling pathway”, “cytokine-cytokine receptor interaction”, “ECM-Receptor interaction”, and other pathways (*P* < 0.05 and q < 0.25) (Supplementary Table S5-S6). The top 5 pathways were selected for visualization based on the order of significance (Figures 5A,B). These pathways and biological processes were intertwined and collectively participate in the onset, progression, and subsequent pathology of AMI. A deeper understanding of the role of these pathways could provide a theoretical basis for the discovery of novel therapeutic strategies in the future.

### Revealing the immune profile in AMI

The development of AMI might be accompanied by abnormalities in the proportion and function of immune cells, so we mined the links between two biomarkers and 22 immune cells. The infiltration content of 22 immune cells in each sample of the GSE62646 dataset was calculated by the CIBERSORT algorithm, as demonstrated in Figure 5C. After removing the cells that turned out to be 0 in 30% of the samples, the expression of the remaining 16 immune cells in AMI and control samples was compared applying the Wilcoxon test, which showed that five immune cells were markedly distinct between groups (*P* < 0.05) (Figure 5D). Among them, M0 macrophages, naive CD4 T cells, and regulatory T cells (Tregs) were more abundant in AMI, while monocytes and neutrophils showed the opposite trend. In addition, the strongest positive association was presented between neutrophils and monocytes (co*r* = 0.53), and the highest negative association was presented between monocytes and M0 macrophages (cor = −0.43) (Figure 5E). Notably, a marked positive association was observed between SNORD54 and Tregs (co*r* = 0.307, *P* < 0.05) (Figure 5F). These findings revealed a specific pattern of immune cell imbalance in AMI and preliminarily established an association between biomarkers and the immune microenvironment.

### Exploration of potential regulatory mechanisms

**Figure 6:**
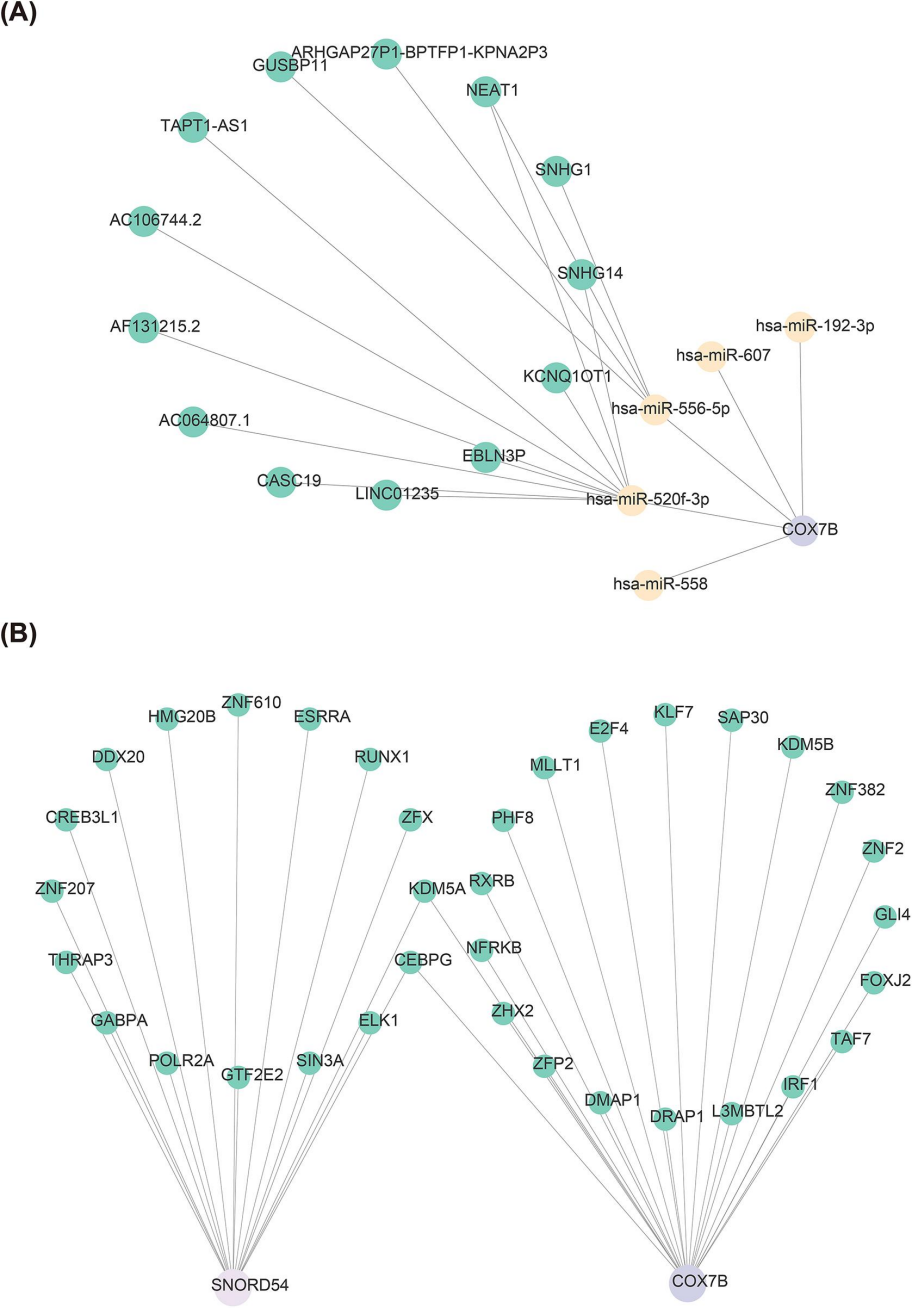
Potential regulatory mechanisms of AMI biomarkers. **(A)** The mRNA-miRNA-lncRNA relationship network of COX7B; **(B)** TF Interaction Network with Candidate Genes.

The mRNA-miRNA-lncRNA relationship network was composed of one mRNA (COX7B), five miRNAs, and 13 lncRNAs (Figure 6A). All five miRNAs (hsa-mir-558, hsa-mir-520f-3p, hsa-mir-556-5p, hsa-mir-607, hsa-mir-192-3p) targeted COX7B; unfortunately, no miRNAs regulating SNORD54 were predicted. In addition, it could be found that lncRNAs (KCNQ1OT1, EBLN3P, LINC01235, etc.) regulation of COX7B was achieved through hsa-mir-520f-3p, as well as NEAT1 could simultaneously regulate COX7B through hsa-mir-520f-3p and hsa-mir-556-5p. In total, 35 TFs were retrieved, including 21 TFs regulating COX7B and 16 TFs regulating SNORD54, and the network of TF-biomarker was presented in Figure 6B. It could be observed that two TFs (KDM5A and CEBPG) could regulate two biomarkers simultaneously. This analysis preliminarily constructed a molecular network framework suggesting that COX7B and SNORD54 may be regulated by non-coding RNAs and transcription factors, thereby laying the groundwork for subsequent mechanistic investigations.

### Biomarkers were associated with m6A regulators

**Figure 7:**
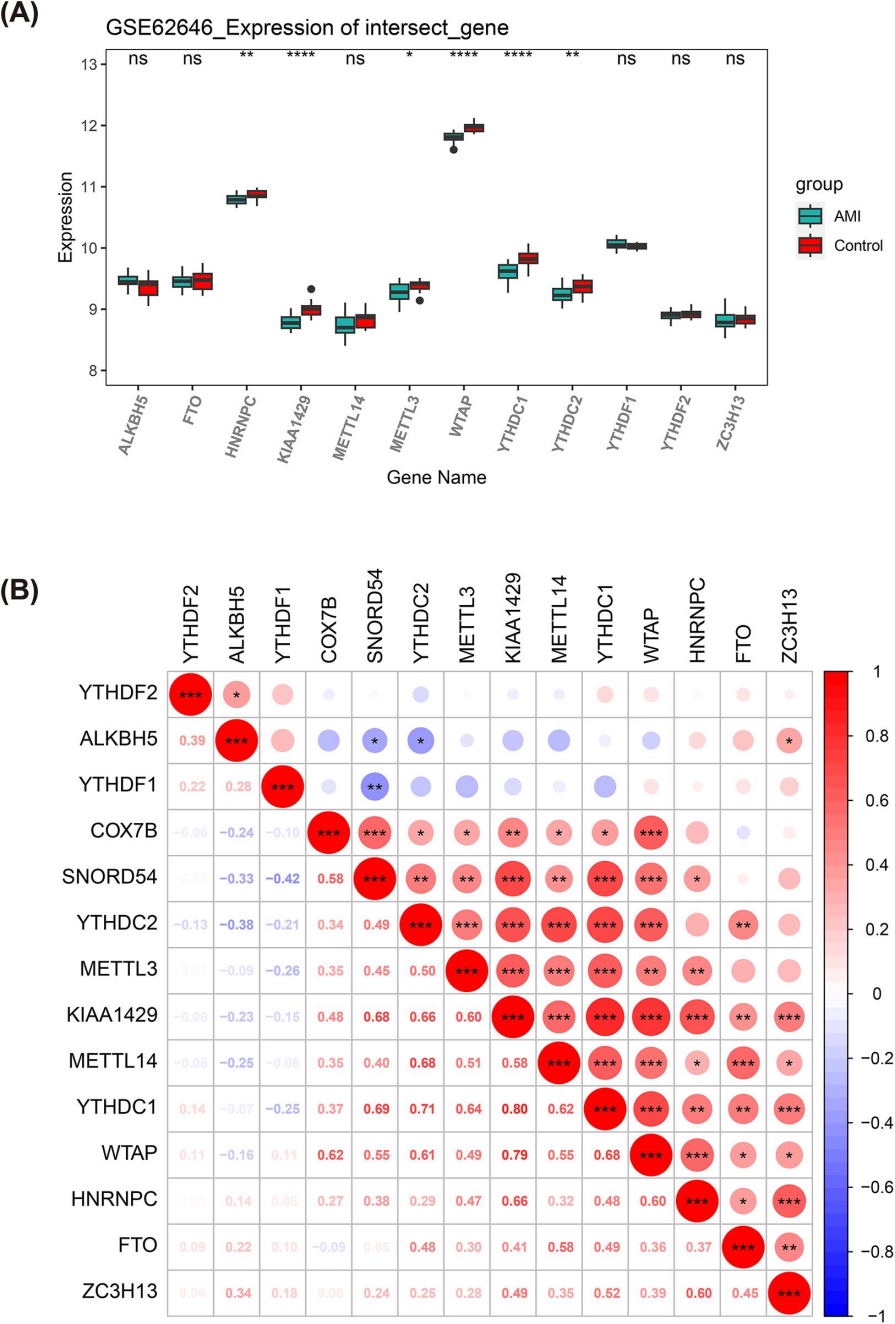
Correlation analysis of m6A regulators. **(A)** Box plots of m6A modification-related gene expression in AMI and control samples in the GSE62646 dataset; **(B)** Heatmap of correlations between m6A modification-related genes and two AMI biomarkers.

The m6A modifications palyed a pivotal role in the regulation of gene expression after AMI and might affect inflammatory responses, apoptosis, and cardiac regenerative repair processes. In this study, six m6A regulators (HNRNPC, KIAA1429, METTL3, WTAP, YTHDC1, and YTHDC2) were found to be significantly different between AMI and control samples in GSE62646 dataset (*P* < 0.05) (Figure 7A), and all of them were under-expressed in AMI samples. Notably, COX7B had the highest marked positive association with WTAP (co*r* = 0.62, *P* < 0.001), and SNORD54 had the strongest significant positive association with YTHDC1 (co*r* = 0.69, *P* < 0.001) (Figure 7B). These correlations suggest that the dysregulation of COX7B and SNORD54 expression may co-vary with the extensive alterations in m6A modification observed in AMI.

### Binding of biomarkers to potential drugs

**Figure 8:**
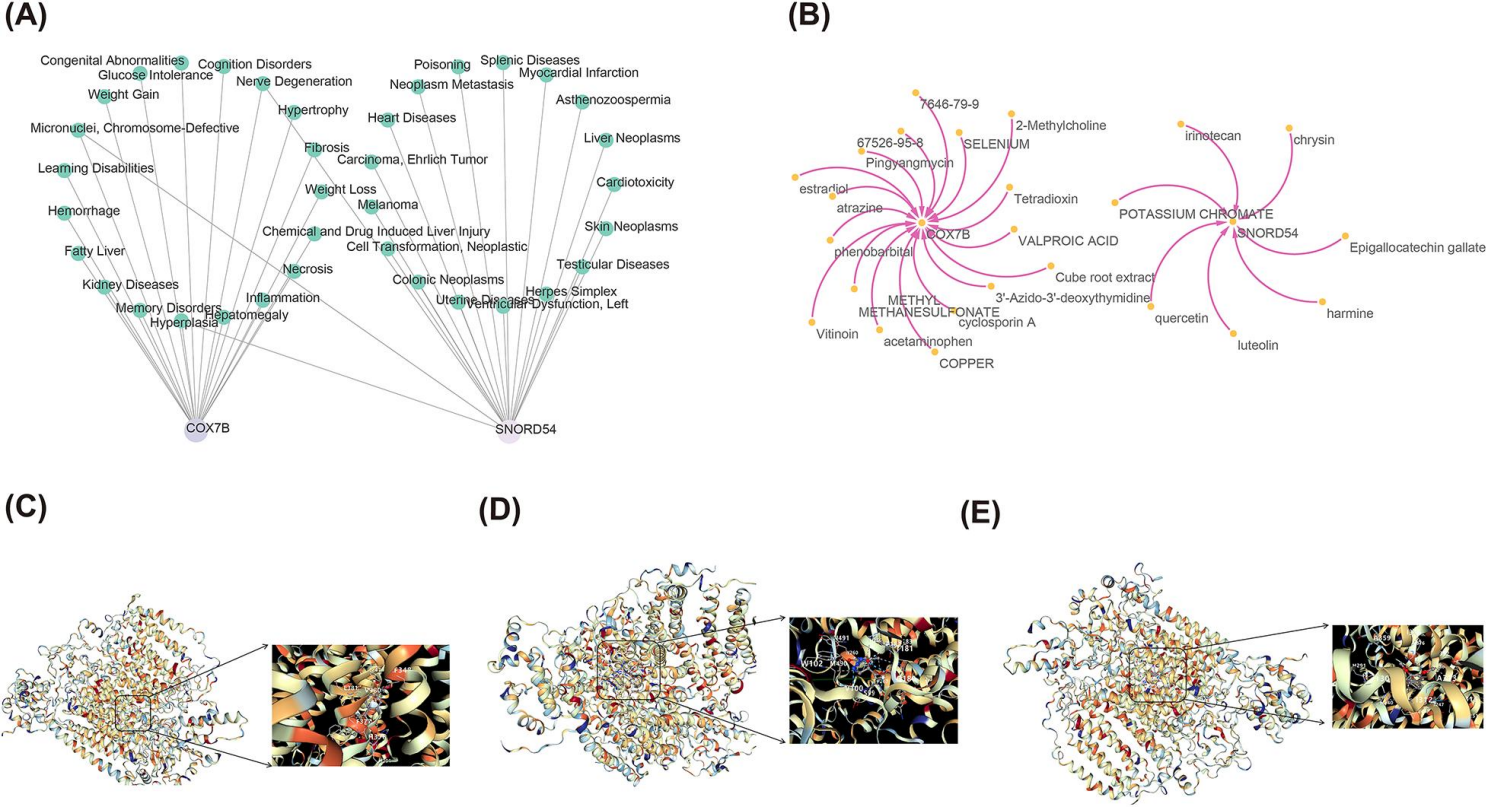
Biological mechanisms of AMI biomarkers. **(A)** Association analysis between two AMI biomarkers and disease; **(B)** Drug prediction network for two AMI biomarkers; **(C–E)** Molecular docking modelling between COX7B's and 3 drugs.

A comprehensive analysis of CTD database was conducted to retrieve diseases significantly linked to the two biomarkers, which revealed that chromosome-defective, nerve degeneration, and hyperplasia could be predicted by both biomarkers (Figure 8A). Additionally, SNORD54 could be found to be significantly associated with cardiovascular diseases such as ventricular dysfunction (left), heart diseases, and myocardial infarction. Further, the prediction of the DGIdb database showed that 25 drugs related to the biomarkers were retrieved, of which the top 3 drugs significantly related to COX7B were 1-Methyl-4-phenyl-2,3-dihydropyridinium, cube root extract, 3'-Azido-3'- deoxythymidine, and the top 3 drugs significantly associated with SNORD54 were chrysin, quercetin, and harmine (Figure 8B). The molecular docking results revealed that the binding energies of COX7B with 1-Methyl-4-phenyl-2,3-dihydropyridinium, cube root extract, and 3'-Azido-3'-deoxythymidine were −8.1 kcal/mol, −7.5 kcal/mol, and −10.9kcal/mol, accordingly, implying that COX7B had a strong affinity for all three drugs (Figures 8C–E). Because SNORD54 had no protein structure, molecular docking was not performed. This section of the results provides preliminary leads for future exploration of compounds targeting COX7B and the potential roles of drugs related to SNORD54 (such as chrysin and quercetin) in AMI.

### Monocytes and NK cells were selected as the key cell type

**Figure 9:**
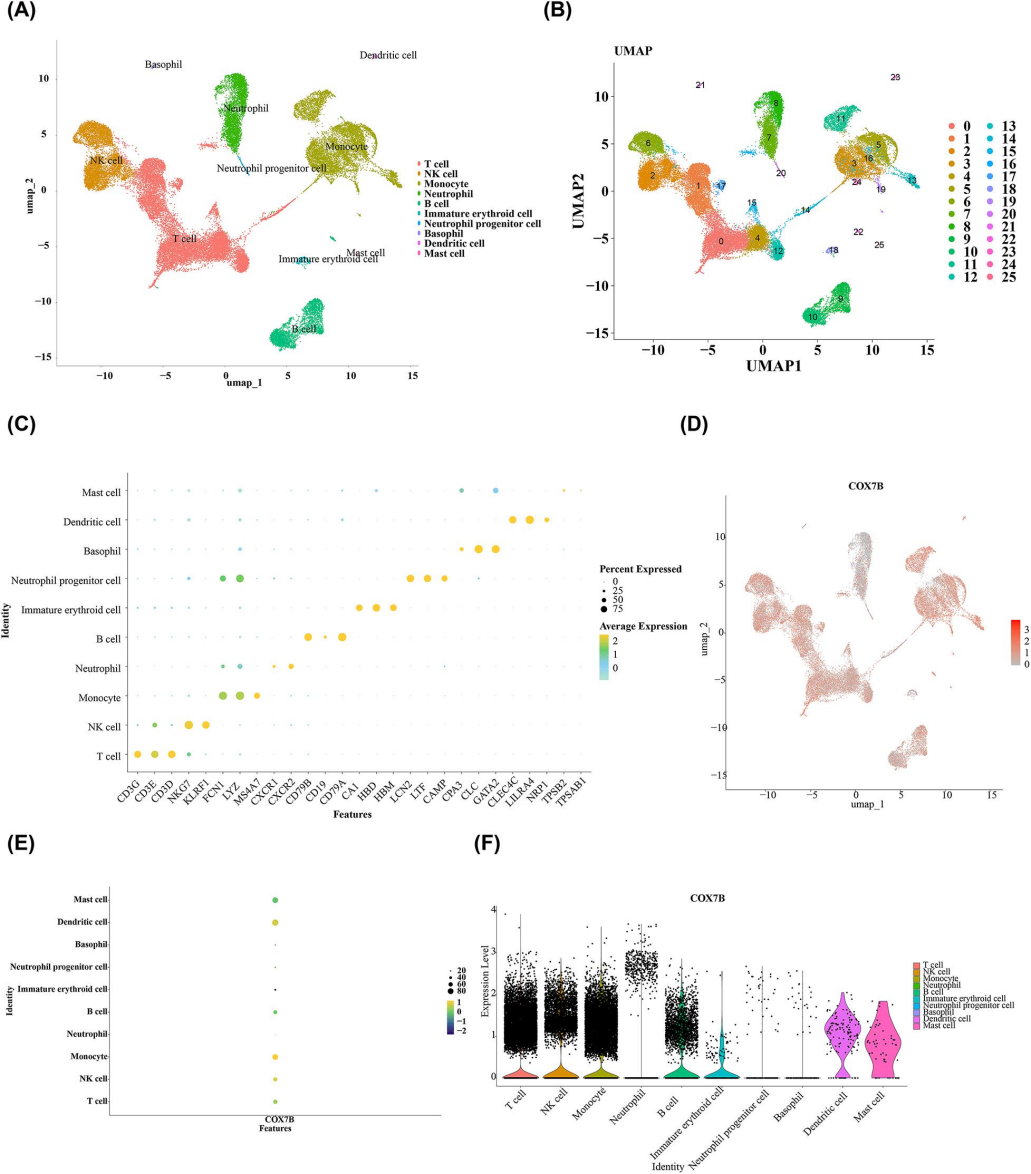
The key cell type of AMI biomarkers. **(A)** The different cell clusters; **(B)** Subtype of cell; **(C)** The bubble plot shows the marker genes exhibited high specificity; **(D–F)** The cells in which the expression levels of biomarkers were relatively high and had large cell number of cells were defined as key cells.

We screened single-cell sequencing data from 5 AMI samples, resulting in a total of 26,957 genes and 33,162 cells. The results before and after QC were showed in Supplementary Figures S1A, S1B. After standard data processing, we selected 2,000 HVGs for subsequently analysis, of which the top 10 genes exhibiting the most pronounced intercellular expression changes were labeled (Supplementary Figure S1C). There were no obvious outlier samples (Supplementary Figure S1D). The top 30 PCs were chosen for further analysis (Supplementary Figure S1E–F). Then, a sum of 26 different cell clusters were identified (Figure 9A), and 26 clusters were annotated to 10 cell types, namely T cells, natural killer (NK) cells, monocytes, neutrophils, B cells, immature erythroid cells, neutrophil progenitor cells, basophils, dendritic cells and mast cells (Figure 9B). The bubble plot demonstrated that the marker genes exhibited high specificity (Figure 9C). Among the two biomarkers, only COX7B existed in the single-cell dataset. Therefore, COX7B was used to search for key cells. According to the results of Figures 9D–F and Table 1, the number of monocytes is large, and the expression level of COX7B in monocytes was the highest. Meanwhile, the number of NK cells was also relatively large, and the expression of COX7B was also relatively high. Therefore, monocytes and NK cells were selected as the key cells.

**Table 1:** Cell type and number.

Celltype	Cell number
Mast cell	55
Dendritic cell	130
Basophil	148
Neutrophil progenitor cell	166
Immature erythroid cell	254
B cell	3,606
Neutrophil	4,323
NK cell	6,361
Monocyte	8,735
T cell	14,689

### Key cells had some communication with a variety of cell types

**Figure 10:**
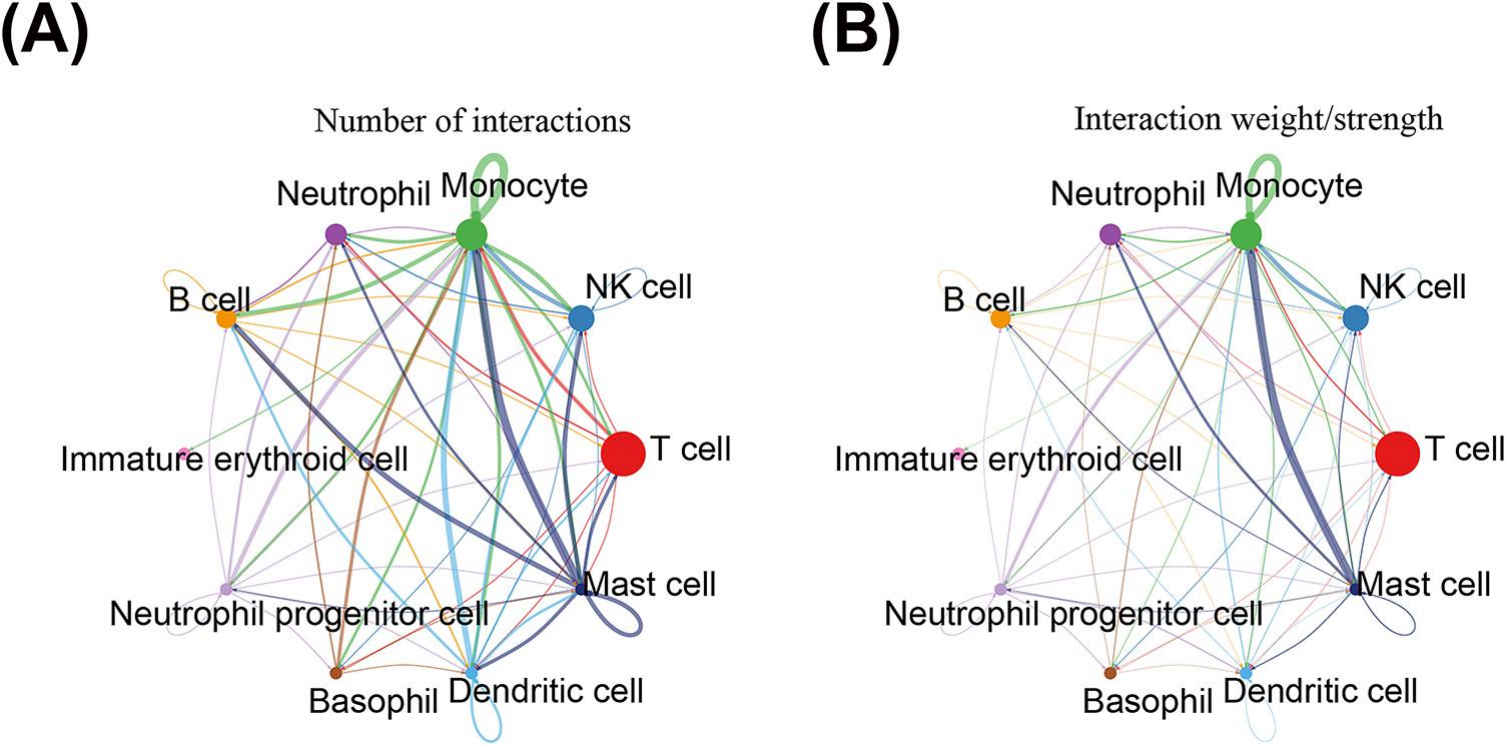
Intercellular communication networks between different cell types in AMI groups. **(A)** The intercellular communication networks anlysis in terms of the number of interactions; **(B)** The intercellular communication networks anlysis in terms of interaction weights.

We conducted an analysis of intercellular communication networks between different cell types in AMI groups. Particularly, all cell types interacted with each other. In terms of the number of interactions, mast cells had the strongest interaction with monocytes, and NK cells had relatively strong interactions with both monocytes and mast cells (Figure 10A). In terms of interaction weights, monocytes also had the strongest correlation with mast cells, and NK cells had the strongest correlation with monocytes (Figure 10B). This suggests that within the AMI microenvironment, monocytes and NK cells exhibit active crosstalk with other immune cells, particularly mast cells, potentially working together to orchestrate the immune response.

### The biomarkers expression levels changed over time with the differentiation of key cells

**Figure 11:**
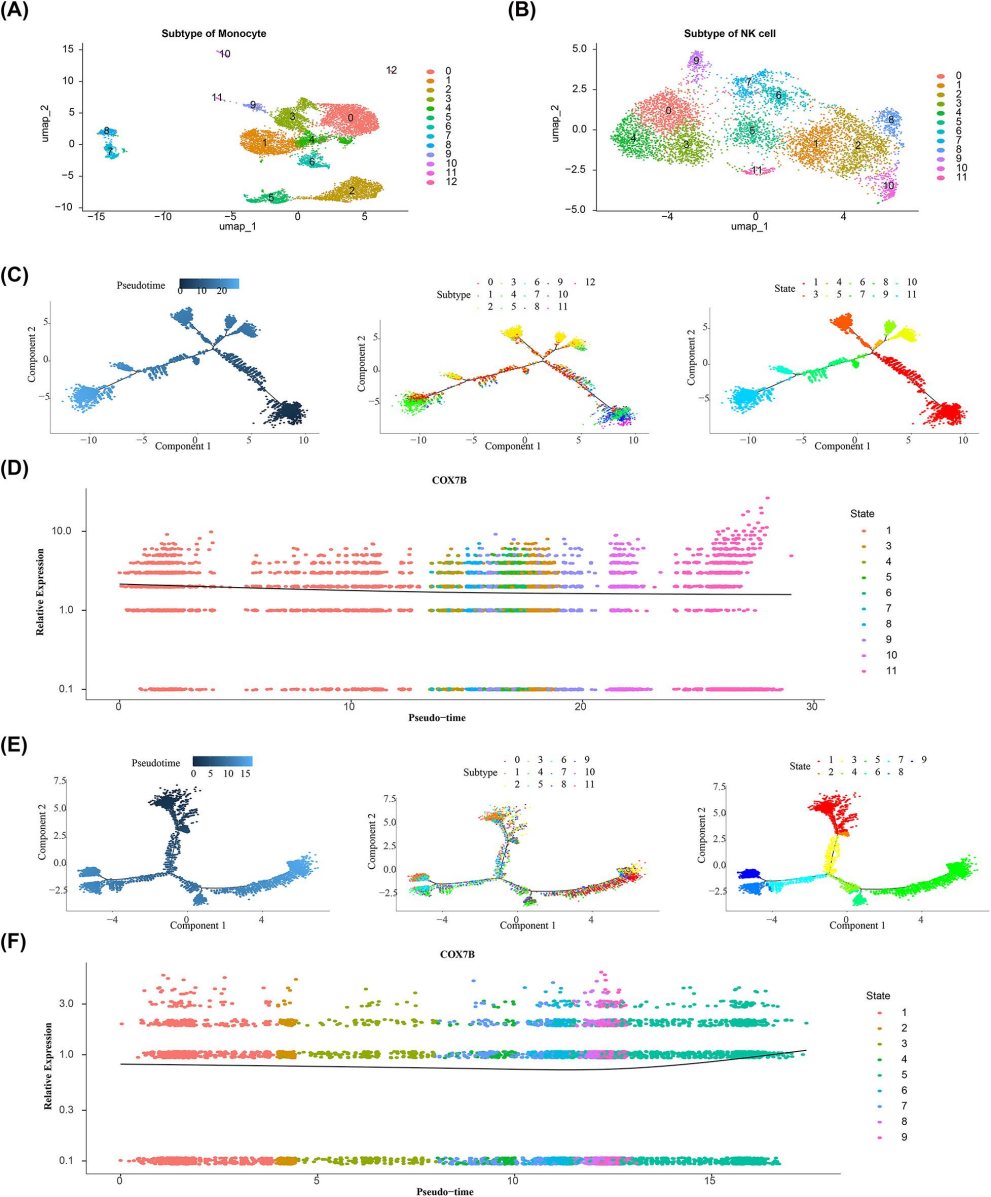
The biomarkers expression levels changed with the differentiation of key cells. **(A,B)** Secondary clustering anlysis for GSE269269; **(C)** The pseudo-time trajectory inference analysis of monocytes; **(D)** The expression of COX7B gene at different stage of monocytes; **(E)** The pseudo-time trajectory inference analysis of NK cells; **(F)** The expression of COX7B gene at different stage of NK cells.

In GSE269269, secondary clustering based on monocytes (resolution = 0.3) and NK cells (resolution = 0.8) was performed, respectively, reclassifying the monocytes into 13 distinct subtypes and the NK cells into 12 distinct subtypes (Figures 11A,B). In addition, in the results of the pseudo-time trajectory inference analysis, the trajectories of monocytes were shown in Figure 11C. Cells differentiate from right to left over time. Monocytes were found to include a total of 10 different states of differentiation and 13 subtypes. For monocytes, state 1 was the early stage of its development. The COX7B gene was highly expressed throughout all states (Figure 11D). Meanwhile, the trajectories of NK cells were shown in Figure 11E. Cells differentiate from up to down over time. NK cells were found to include a total of 9 different states of differentiation and 12 subtypes. For NK cells, state 1 was the early stage of its development. The COX7B gene was also highly expressed throughout all states (Figure 11F). Pseudotime analysis indicated that COX7B maintained consistently high expression across different differentiation stages and subtypes of monocytes and NK cells, suggesting that its role in the basic functions of these immune cells may be important in the context of AMI.

## Discussion

In this study, we employed the LASSO and Boruta algorithms to screen DEGs, identifying COX7B and SNORD54 as mitochondrial dynamics-related biomarkers in AMI through ROC validation. Both genes exhibited significant down-regulation in AMI compared to controls. Subsequent analyses encompassing GSEA, immune infiltration profiling, and drug prediction, complemented by scRNA-seq, revealed monocytes and NK cells as pivotal contributors to AMI pathogenesis. These findings advance our understanding of AMI mechanisms and propose novel therapeutic and diagnostic strategies.

COX7B (Cytochrome C Oxidase Subunit 7B), a critical subunit of cytochrome c oxidase (Complex IV) in the electron transport chain, demonstrates context-dependent roles in cardiovascular diseases. While bioinformatics studies implicate COX7B in atherosclerosis progression—a precursor to AMI through vessel wall thickening and luminal narrowing—its expression dynamics vary across disease stages (8). COX7B expression is positively correlated with cardiomyocyte size. In cardiac hypertrophy, COX7B upregulation compensates for early metabolic stress but declines during heart failure (34). We observed reduced COX7B expression in AMI, which contrasts with its transient upregulation in cardiac hypertrophy. This divergence may reflect fundamental differences in disease progression (acute ischemia vs. chronic remodeling), cellular adaptation thresholds, or sample heterogeneity. This divergence underscores the necessity for stage-specific investigations into COX7B's role, particularly its interplay with mitochondrial oxidative phosphorylation (OXPHOS) and apoptosis regulation.

SNORD54 (Small Nucleolar RNA, C/D Box 54), as a non-protein-coding small nucleolar RNA that belongs to the C/D box class of small nucleolar RNAs (snoRNAs), SNORD54 modulates ribosomal RNA 2'-O-methylation, a process critical for ribosome maturation (35). Emerging evidence links ribosomal dysfunction to metabolic disorders and aging—key risk factors for AMI (36, 37). SNORD54, as one of the key factors in ribosome biosynthesis, may play an important role in the occurrence and development of AMI. Our clinical validation of SNORD54 down-regulation in AMI aligns with ribotoxic stress models, where impaired rRNA processing exacerbates mitochondrial ROS production and cellular injury (37). Although counterintuitive to assumptions of compensatory snoRNA upregulation during stress, this suppression may reflect exhaustion of ribosomal quality control mechanisms in acute ischemia. Further studies should delineate whether SNORD54 loss directly impairs cardiac ribosome function or serves as a bystander marker of systemic ribotoxic stress.

To identify the key genes COX7B and SNORD54, we performed cross-validation by integrating two independent datasets, GSE62646 and GSE59867, to strengthen the robustness of our findings. During the analysis, we observed that some candidate genes showed significant expression differences and high diagnostic performance (AUC > 0.7) in the GSE62646 dataset, but their expression trends or diagnostic efficacy did not reach comparable significance in GSE59867. This heterogeneity between datasets may arise from several factors. First, differences in cohort sample sizes could affect statistical power and stability. Second, variations in demographic characteristics, comorbidities, medication use, as well as subtle differences in sample collection and processing protocols, may introduce technical or biological variability that influences gene expression profiles. Furthermore, despite efforts to control analytical conditions, inherent batch effects across studies could also challenge the comparability of gene expression between datasets.

This observation underscores the necessity of validation in independent, large-scale, and multi-center cohorts. It reminds us that biomarkers identified from a single dataset may have limited generalizability to broader populations. Therefore, we ultimately focused on COX7B and SNORD54, which exhibited consistent expression and stable diagnostic performance across both datasets, thereby mitigating limitations associated with reliance on a single cohort. Future studies should validate these genes in larger, multi-center populations to further enhance the reliability of our findings and their potential for clinical translation.

GSEA analysis from the GSE62646 dataset highlighted OXPHOS and MAPK signaling as central pathways associated with both biomarkers. Mitochondrial ROS, a byproduct of OXPHOS imbalance, drives endothelial dysfunction and cardiomyocyte apoptosis in ischemia-reperfusion injury (38). Notably, strategies to mitigate mitochondrial ROS (e.g., enhancing antioxidant defenses) improve coronary endothelial resilience and post-infarct outcomes (39). Concurrently, MAPK hyperactivation—observed here and in prior studies—exacerbates ER stress and apoptosis via p38/JNK signaling (40). Intriguingly, alkaloids targeting p38 MAPK (e.g., Corydalis hendersonii derivatives) attenuate ischemic injury by restoring mitochondrial-ER crosstalk (41), suggesting COX7B/SNORD54 may similarly influence MAPK dynamics. These intersecting pathways position both biomarkers as potential nodes for therapeutic intervention.

Given the crucial role of the immune system in the pathophysiology of acute myocardial infarction (AMI), our study revealed distinct immune cell infiltration profiles in AMI through CIBERSORT analysis. Compared with controls, AMI patients showed a significant decrease in the proportions of CD8^+^T cells and resting mast cells in peripheral blood, along with markedly increased infiltration of neutrophils and M0 macrophages. This suggests that the imbalance of these immune cell subsets may contribute to the pathological process of AMI. Further single-cell RNA sequencing (scRNA-seq) analysis identified monocytes and natural killer (NK) cells as key cell types mediating AMI pathophysiology.

In addition, correlation analysis between biomarkers and immune cells indicated that COX7B expression was positively correlated with M0 macrophages, while SNORD54 was positively correlated with regulatory T cells (Tregs). These associations suggest that COX7B and SNORD54 may be involved in the development of AMI by regulating specific immune cell functions. As central pleiotropic cells of the innate immune system, monocytes and macrophages play an indispensable role in initiating the early inflammatory response and subsequent wound healing after cardiac injury (42). We therefore speculate that altered expression of COX7B may influence the activation or functional phenotype of M0 macrophages, thereby modulating the balance between inflammatory response and tissue repair following AMI. Tregs, an immunosuppressive T-cell subset, can reduce infarct size, improve cardiac function, and attenuate pathological remodeling through mechanisms such as suppressing pro-inflammatory cell recruitment, modulating macrophage phenotypic switching, mitigating myocardial fibrosis, protecting cardiomyocyte survival, and promoting angiogenesis (43). The positive correlation between SNORD54 and Tregs implies that SNORD54 may help maintain Treg functional stability and contribute to the regulation of immune homeostasis after AMI.

Furthermore, NK cells, as important innate immune effectors, secrete inflammatory cytokines such as IFN-*γ* and are not only involved in the pathogenesis of AMI but also participate in regulating pathological cardiac remodeling after infarction (44). Their identification as key cells further supports the central role of immune cells in AMI pathology. Future research could focus on immune-related pathways to explore how key genes influence AMI through immune cell regulation, thereby providing a rationale for immune-based therapeutic strategies in AMI.

Our findings also intersect with the roles of long non-coding RNAs (lncRNAs) and N6-methyladenosine (m6A) modifications, which are emerging as critical regulators in AMI. NEAT1 suppression in AMI disrupts paraspeckle formation, potentiating inflammatory cytokine release (45, 46), while the KCNQ1OT1/miR-26a-5p/ATG12 axis aggravates autophagic flux dysregulation (47). Our network analysis positions COX7B/SNORD54 within these circuits, possibly via m6A-dependent mechanisms. Strikingly, WTAP—an m6A “writer” that promotes ER stress and apoptosis by stabilizing ATF4 mRNA—showed strong co-expression with COX7B (48). This nexus implies WTAP-mediated m6A modifications may modulate COX7B's mitochondrial roles, offering a translational bridge between epigenetic regulation and metabolic adaptation.

Cardiac energy demands render mitochondrial quality control indispensable. While transient autophagy protects during ischemia by clearing damaged mitochondria, excessive mitophagy during reperfusion exacerbates injury (49, 50). Our findings align with this biphasic paradigm: COX7B/SNORD54 downregulation may impair mitochondrial fission-fusion balance, triggering ROS-driven inflammation and apoptosis (51). Targeting these biomarkers could fine-tune mitochondrial dynamics, potentially circumventing the pitfalls of global autophagy modulation.

Although our study validated the transcriptional changes of COX7B and SNORD54 in blood samples using RT-qPCR and confirmed their diagnostic potential as biomarkers for acute myocardial infarction (AMI), several limitations remain to be addressed. First, during the bioinformatics analysis stage, we used patients with stable coronary artery disease and no history of myocardial infarction as controls, whereas the subsequent experimental validation employed samples from healthy individuals. Although this difference in control groups helps assess biomarker specificity from different perspectives, it also limits the direct comparability and interpretative consistency of the results within a unified disease spectrum. In addition, the relatively limited sample size may affect statistical power and generalizability, underscoring the need for validation in larger, multi-center cohorts with well-defined and uniform control designs.

Second, the current validation of these biomarkers remains primarily at the transcriptional level, lacking direct evidence at the protein level. Finally, this study largely relies on bioinformatics analysis and public datasets, with a lack of systematic *in vivo* or *in vitro* functional validation. Consequently, it remains difficult to determine the causal role of COX7B and SNORD54 in AMI development or their dynamic expression patterns, and the proposed mechanistic insights and drug predictions remain hypothetical, requiring rigorous verification before clinical translation can be considered.

To address these limitations, future research should expand the sample size and employ a stratified control design, including healthy controls, stable coronary artery disease patients, and AMI patients, in an independent validation cohort. Furthermore, multi-level and multi-omics functional investigations combining animal models and cell experiments are needed. These should include confirming their expression and modification at the protein level, such as through Western blot or immunohistochemistry in clinical tissue samples or animal models of myocardial infarction, to further elucidate their protein expression patterns and cellular localization. Gain-or loss-of-function experiments should also be conducted to clarify their direct effects on mitochondrial dynamics, ROS generation, and cardiomyocyte apoptosis. Through such approaches, the translational value of COX7B and SNORD54 as early diagnostic markers, risk-stratification tools, and potential therapeutic targets can be systematically evaluated.

## Conclusions

In conclusion, this study establishes COX7B and SNORD54 as dual biomarkers with clinical translational potential in AMI, crucially associated with mitochondrial homeostasis. Through scRNA-seq analysis, we delineated monocytes and NK cells as pivotal cellular mediators in AMI pathophysiology. Our work bridges mitochondrial dynamics with immune dysregulation in myocardial injury. These findings provide a novel molecular framework for developing precision diagnostic tools and therapeutic strategies. Future investigations should focus on validating their utility in early disease detection, prognostic stratification, and as novel therapeutic targets to refine clinical management paradigms for AMI.

## Data Availability

The datasets presented in this study can be found in online repositories. The names of the repository/repositories and accession number(s) can be found in the article/Supplementary Material.
