# Supplementary material for: Uncovering mitochondrial dynamics–related genes as potential diagnostic biomarkers for acute myocardial infarction
Source: Front Cardiovasc Med. 2026 Feb 13;13:1755024. doi: 10.3389/fcvm.2026.1755024 (PMC12946055; doi:10.3389/fcvm.2026.1755024)
Supplement: Supplementary file 1 [file Datasheet1.docx]

**Supplementary information**

**Supplementary Table**

Table S1 List of mitophagy-related genes

Table S2 Primer sequence list

Table S3 GO enrichment analysis list of differentially expressed genes

Table S4 KEGG enrichment analysis list of differentially expressed genes

Table S5 GSEA analysis results (COX7B)

Table S6 GSEA analysis results (SNORD54)

**Supplementary Figure**

**
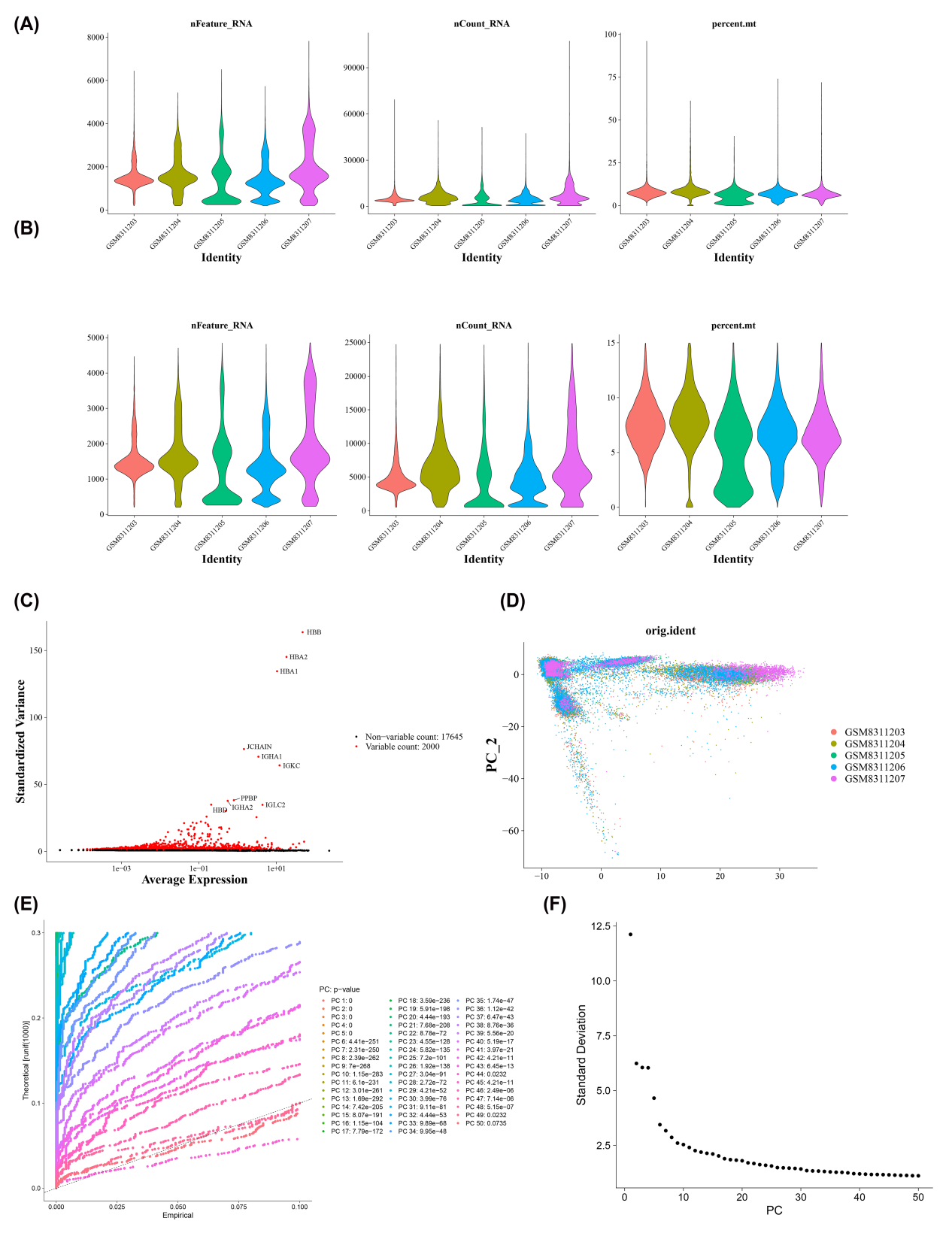
**

**Figure S1 scRNA-seq analysis for 5 AMI samples. (A-B)** Violin plot shows The 5 AMI samples before and after QC; **(C)** The highly variable genes After standard data processing and the top 10 genes were labeled; **(D)**There were no obvious outlier samples; **(E-F)** The top 30 PCs.
