# Supplementary material for: Uncovering mitochondrial dynamics–related genes as potential diagnostic biomarkers for acute myocardial infarction
Source: Front Cardiovasc Med. 2026 Feb 13;13:1755024. doi: 10.3389/fcvm.2026.1755024 (PMC12946055; doi:10.3389/fcvm.2026.1755024)
Supplement: Supplementary file 2 [file Table2.docx]

Table S2 Primer sequence list

| **Primers** | **Sequences** | |
| --- | --- | --- |
| COX7B-F | GCAGAGCCACCAGAAACGTA | |
| COX7B-R | ACAGGGGACAGGTTCCATTC |  |
| SNORD54-F | CGATGAGGAGGTACCTATTGT |  |
| SNORD54-R | GGCTCAGAATAGCGTATAAAA |  |
| GAPDH-F | CGAAGGTGGAGTCAACGGATTT |  |
| GAPDH-R | ATGGGTGGAATCATATTGGAAC |  |
